# Account of a Case in Which the Colour of the Skin Was Changed

**Published:** 1786

**Authors:** M. de Chemseru


VI. *Account of a Caſe in which the Colour of the Skin was changed. By M.* de Chamſeru.—Vide *Hiſtoire de la Société Royale de Medecine*; Années 1780 & 1781. 4to. Paris, 1785.

THE title* which M. de Chamſeru has thought proper to prefix to this hiſtory, though it accords with his theory of the diſeaſe, is very far from conveying an idea of what we conſider as the moſt curious circumſtances that accompanied it.

The caſe is that of a young lady, for whom the author was conſulted in the year 1778. The patient was then eight years old. The account given of her by her father was, that ſhe was born with a very uncommon colour of the ſkin; that her face in particular, and her upper and lower extremities, were, from her birth, of a violet complexion, which, by degrees, uniformly extended itſelf over the other parts of her body.

This colour, at the time ſhe was brought to M. de Chamſeru, was ſo deep, that her lips, cheeks, tongue, the whole of the inſide of her mouth, and the tips of her fingers, were almoſt

* Obſervation ſur un Changement de Couleur de la Peau.

black.

black. The tunica conjunctiva of both eyes had likewife acquired a violet tinge, which was particularly remarkable at the iris.

Her hair was of a brown colour; her countenance, from the coarfenefs of her features, as well as from the peculiarity of her complexion, had a confiderable refemblance to that of a mulatto. Her eyes were prominent; her nofe a little flattened at its upper part; the noftrils were wide; and fhe had a large mouth, and thick lips.

Her height was lefs than might have been expected at her age. Judging from her ftature only, the author fays, he fhould have fuppofed her to have been not more than five or fix years old. Her fhoulders were elevated; her abdomen was fomewhat larger than might have been expected confidering her age and ftature, but was foft and feemingly free from obftruction. Her arms, thighs, and legs, were flender, but without any appearance of rickets.

This child, her father faid, had been with difficulty reared, though fhe had never experienced any dangerous difeafe. She had always been very weak in her limbs, and walked with difficulty. Her breathing was conftantly laborious, and the leaft degree of motion, even the

 act act of chewing or of ſwallowing her food, was ſufficient to increaſe this difficulty of reſpiration. It uniformly grew worſe after eating; and every meal, though ſhe ate but little, was ſure to be followed by apparent danger of ſuffocation, accompanied ſometimes by a violent cough, and vomiting.

She breathed more freely in an horizontal poſture than when ſtanding or ſitting; and, on this account, ſhe had for ſome time been accuſtomed to remain in bed ſeveral hours after dinner: nevertheleſs her ſleep was never perfectly eaſy, and ſhe conſtantly waked ſeveral times in the night, eſpecially when a ſnoring, to which ſhe was ſubject, increaſed to ſuch a degree as to indicate danger of ſuffocation. Whenever this recurred, her friends were in the habit of giving her a draught of cold water, which generally had a good effect.

Her pulſe was weak, quicker than natural, and ſometimes intermittent.

When ſhe roſe in the morning, her ſkin was conſtantly obſerved to have loſt much of its violet complexion, and on her breaſt and abdomen ſeemed to have become almoſt white. Her face and extremities were of a deeper colour; but her cheeks, lips, tongue, gums, and the ends of her fingers, inftead of appearing of a blackifh tinge, were, at this part of the day, more inclined to a red hue : but in the fpace of about two hours after fhe was up, the dark colour gradually returned.

It is to be regretted that the fequel of the cafe is not known, the patient's father having taken her with him from Paris foon after our author was confulted, and all attempts to procure a farther account of her fince that time having proved unfuccefsful.

M. de Chamferu, from the colour of the fkin in this cafe, was induced to confider the difeafe as a fpecies of icterus; but when, to the general appearance of the fkin, we add the particular deepnefs of its tinge in the face and extremities, the difficulty of refpiration, the irregular ftate of the pulfe, the general weaknefs of the patient, and her inability for mufcular exertion, together with the flendernefs of her limbs, and compare thefe and the other circumftances of the cafe with the inftances we have collected * of malconformation of the

* See Vol. VI. pages 407, 433, 434, and 436; and likewife the fubject of the next article, which completes our collection of the inftances hitherto publifhed of this malconformation.

heart, we do not heſitate to aſcribe them to a ſimilar cauſe.

M. de Chamſeru ſpeaks of a phyſician, who, on being conſulted for this patient, obſerved, that in the courſe of near fifty years practice he had met with only one inſtance of a diſeaſe ſimilar to this, and that in a boy, ſeven years old, who had exactly the ſame ſymptoms as this girl. He conſidered it, we are told, as a diſeaſe analogous to ſcurvy, and treated it accordingly, but the patient died in about two months. — The ſame phyſician, M. de Chamſeru adds, has ſince ſeen another child labouring under a ſimilar complaint, for which he has likewiſe adviſed an antiſcorbutic regimen; but of the particular circumſtances of this caſe no account is given.

